# The teaching order of using direct laryngoscopy first may improve the learning outcome of endotracheal incubation: A preliminary, randomized controlled trial

**DOI:** 10.1097/MD.0000000000015624

**Published:** 2019-05-24

**Authors:** Minglu Gu, Ming Lian, Chao Gong, Lianhua Chen, Shitong LI

**Affiliations:** a Department of Anesthesiology, Shanghai General Hospital, Shanghai Jiaotong University; b Xinhua Hospital Affiliated to Shanghai Jiaotong University School of Medicine, Shanghai, China.

**Keywords:** direct laryngoscopy, endotracheal intubation, intubation time, learning curve, video laryngoscopy

## Abstract

**Background:**

Endotracheal intubation (ETI) is a life-saving procedure taught to medical students. We examined the influence of the order of teaching ETI through direct laryngoscopy (DL) and video laryngoscopy (VL) on learning by measuring the intubation time and learning curve of trainees, in order to explore ways to improve ETI performance.

**Methods:**

Twenty trainees were randomly divided into 2 groups. In the DL-first group, trainees used DL to perform ETI 10 times and then used VL 10 times, while the order was reversed in the VL-first group. Intubation time, number of intubation attempts, the Cormack-Lehane (CL) classification, and adverse events were recorded. The primary outcome was the cumulative summation (CUSUM). The CUSUM equation is defined as 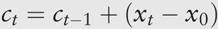, where *c*_*t*_ is the cumulative sum.

**Results:**

ETI was attempted on 400 patients. The difference in the mean times for the first 10 intubations between the 2 groups was not significant (*P* > .05). Mean intubation time for second series in the DL-first group was significantly shorter than that of the first series (*P* < .05), while there were no differences between the 2 series in the VL-first group (*P* > .05). The mean intubation time in the second series of the DL-first group was shorter than for the first series of the VL-first group (*P* < .05), while the mean intubation time of the first series by the DL-first group did not differ from the second series by the VL-first group (*P* > .05). Eighteen attempts were required to achieve an 80% intubation success rate for the DL-first group, while more than 20 attempts were required for the trainees in the VL-first group.

**Conclusion:**

We consider that teaching trainees DL for tracheal intubation first.

**Clinical trial number:**

ChiCTR-OOR-16008364.

## 1 Introduction

Endotracheal intubation (ETI) is a life-saving procedure and one of the most important basic clinical skills in anesthesiology.^[1,2]^ A failed or difficult intubation is an important cause of morbidity and mortality, associated with direct airway trauma and hypoxia.^[3]^ ETI with direct laryngoscopy (DL) is the fastest, most cost-effective airway management method for most patients,^[4]^ and learning ETI with DL is essential in anesthesia and emergency medicine.

During intubation training with DL, the traditional instructor-trainee relationship involves blind verbal feedback to the trainee or the instructor looking over the trainee's shoulder to share the view of the airway. The restricted ability to share the trainee's view of the patient's airway with the instructor is an important teaching limitation.^[4]^

In an attempt to improve the quality of trainee education and patient safety, using video laryngoscopy (VL) has been suggested as a teaching tool for ETI.^[5–8]^ VL, which functions similarly to DL, enables the instructor to guide the trainee via a video monitor, avoiding the drawbacks of DL teaching and directly seeing what the trainee is actually seeing.^[9]^

Even though many studies have compared the teaching effects of the 2 laryngoscopies,^[5–8]^ no studies have reported whether there is an interaction between DL and VL as teaching tools, that is, whether using DL or VL first could enhance the teaching quality of ETI. Therefore, we examined the influence of the order of DL and VL during teaching on the intubation time and the learning curve of ETI. The results could help improve the quality of the teaching of ETI for trainees, as well as the design of more efficient teaching protocols.

## 2 Subjects and methods

### 2.1 Trainees and patients

After institutional ethics committee approval, this preliminary, randomized controlled study was conducted in a tertiary hospital (Shanghai General Hospital, Shanghai Jiao Tong University School of Medicine) between April 2016 and February 2017. Trainees who had no experience with ETI were randomly divided into 2 groups at 1:1 ratio by drawing lots. All trainees were from the same class and university. In the DL-first group, each trainee used DL to perform 10 tracheal intubations and then used VL to perform another 10. In the VL-first group, the 2 series were reversed.

Participation in this study was voluntary and anonymous. All the procedures are in compliance with the Ethics Committee of Shanghai General Hospital Affiliated to Shanghai Jiao Tong University and the National Institute of Health Guide for the clinical research. This study has received the Institutional Ethics Committee approval (No. 2016KY110) of a tertiary-care university hospital (Shanghai General Hospital, Shanghai Jiao Tong University School of Medicine).

Written informed consent to participate in the study was obtained from both patients and trainees. The anesthesiologist assigned elective surgery patients to the trainees for premedication and intubation. Patients were excluded from the study if they had American Society of Anesthesiologists (ASA) physical status class III or greater, or if an unexpectedly difficult ETI occurred. The anesthesiologist assessed the airway conditions using the thyromental distance, inter-incisor gap, modified Mallampati classification, and neck movement, and ruled out those who might have difficult airways.^[10,11]^

### 2.2 Teaching protocol

First, all trainees were given a formal course on ETI. During this course, they were assigned readings, performed human-simulator-based training exercises, and observed clinical anesthesia care (each trainee was exposed glottis with a visual laryngoscope. The trainee was asked to observe the glottis structure and explain the intubation process). On the first 2 days of rotation, all students received basic instruction in airway management procedures, including human-simulator-based training in mask ventilation and ETI. Under the direction of 2 instructors, 4 simulator-based ETI training sessions were held over a 5-day period at the hospital's simulation center. Each trainee practiced with a manikin under an instructor's supervision until a successful ETI using laryngoscopy was performed.

The attending anesthesiologists selected the size of the ETI tube and the laryngoscope blade according to each patient. The patient's head was put in the sniffing position during anesthesia induction and ETI. Mask ventilation was applied during standardized anesthesia induction with 0.05 to 0.075 mg/kg midazolam, 2 to 3 μg/kg fentanyl, and 1.5 to 2.5 mg/kg propofol. A muscle relaxant (0.6–1.2 mg/kg rocuronium) was administered after the patient fell asleep. ETI was performed after complete muscle relaxation. External laryngeal manipulation or the backward upward rightward pressure maneuver was performed, as appropriate. All patients had ETI performed by trainees, with an attending anesthesiologist present and providing ongoing supervision. All training procedures were conducted by 2 senior attendants.

### 2.3 Data collection

In addition to the baseline characteristics, the following intubation-related data were recorded: intubation time, number of intubation attempts, laryngoscopic view using the Cormack-Lehane (CL) classification, and adverse events (defined as desaturation [peripheral oxygen saturation <90%] and bradycardia [heart rate ≤60 bpm]).^[3,12]^ All data were collected immediately after ETI on paper case report forms and were later input into a spreadsheet. Intubation time was defined as the time from insertion of the blade between the teeth to the endotracheal tube was placed in the trachea. Intubation time was not recorded until the completion of a successful intubation. Trainees were allowed up to three attempts to intubate a patient before their supervisor took over. Successful intubation was confirmed by capnography. A failed intubation was recorded if the ETI was not placed in the trachea, the trainee was stopped due to potential harm to the patient, 3 attempts were made in a patient, or 150 s had elapsed.^[13,14]^ The primary outcome was the cumulative summation (CUSUM).

### 2.4 Cumulative summation and risk-adjusted calculation

The primary outcome was the cumulative summation (CUSUM). CUSUM is a control chart statistical method, which has been widely accepted as an objective evaluation standard in the medical field in recent years. The CUSUM equation is defined as 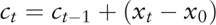, where *c*_*t*_ is the cumulative sum.

The CUSUM was calculated using the formulas: a = ln{(1−*β*)/α)}, b = ln{(1 − α)/*β*}, *P* = ln (*p*1/*p*0), *Q* = ln{(1 – *p*_0_)/(1 − *p*_1_)}, S = Q/(P + Q), *h*_0_ = b/(P + Q), *h*_1_ = a/(P + Q), *n* = {*h*_0_ × (1 − α)−α × *h*_1_}/(S − *p*_0_), and *m* ={*h*_1_ × (1 − *β*) − *β* × *h*_0_}/(*p*_1_ − S), where *p*_0_ is the acceptable failure rate, *p*_1_ is the unacceptable failure rate, α is the probability of wrongly evaluating an trainee's performance as unacceptable or type I error rate, β is the probability of wrongly certifying a trainee's performance as acceptable or type II error rate, *n* is the expected number of attempts to cross the lower decision limit (*h*_0_) under a given failure rate *p*_0_, and *m* is the average number of attempts to cross the upper decision limit (*h*_1_) under a given failure rate *p*_1_. A risk score was calculated for each patient as the estimated probability of failure predicted as the risk factors for difficult intubation using logistic regression. A risk-adjusted CUSUM chart was calculated by adding 1 minus the individual patient risk score to the cumulative score for each failure and subtracting the risk score for each failed attempt. Then, the CUSUM at time *t* (*c*_*t*_) is *c*_*t*_ = *c*_*t*–1_+ (*x*_*t*_–*x*_*0*_), where *c*_*t*–1_ is the CUSUM through the previous attempt, *x*_*t*_ is 1 for failure and is 0 for success (observed), and *x*_*0*_ is the estimated risk of the patient being intubated.^[15,16]^ The limitation of the standard CUSUM method is that it does not allow weighting of the CUSUM score based on the expected difficulty of each procedure, so we set the target success rate for the first intubation at 98%, based on the experience of the senior doctors. Therefore, the inherent failure rate and risk factors, including external laryngeal pressure, CL classification, and intubation-related adverse events, were both defined as 2%.^[17,18]^

### 2.5 Sample size calculation

The sample size for trainees was calculated using the formulas: *n =* (Z_1–α/2_/δ)^2^ × *p* (1 – *p*), where Z, δ and *p* were set to 1.64, 20%, and 50%, respectively. We assumed that at least 16 trainees should be included in the study, with at least 8 trainees in each group.

The sample size for minimal practice procedure was calculated based on the CUSUM calculation, an acceptable failure rate (*p*_0_) of 20%, and an unacceptable failure rate (*p*_1_) of 40%. The type I (α) and type II (β) errors were set to 0.1. The expected number of attempted procedures to cross *h*_0_ and average number of attempted procedures to cross *h*_1_ were 19 and 17, respectively. We assumed that the trainees would each attempt 20 ETI procedures during their 2 weeks of training.

### 2.6 Statistical analysis

Categorical variables were compared using the chi-square or Fisher exact test, as appropriate. Continuous variables were analyzed using the Student's *t* test or Wilcoxon rank-sum test and expressed as means ± standard deviation (SD). Standard CUSUM and risk-adjusted CUSUM were calculated using the formula shown above. Both standard and risk-adjusted CUSUM charts were plotted for each trainee. Multivariate analyses were performed by fitting a logistic regression model that included all variables with a *P* < .05 in the univariate analyses.^[15]^ Two-sided *P* < .05 was considered as statistically significant.

## 3 Results

### 3.1 Baseline characteristics

The study enrolled 20 trainees and 400 patients, with 10 trainees and 200 patients in each group. Each trainee completed 20 intubation attempts within 2 weeks. Baseline characteristics are summarized in Table 1. Age, gender, body mass index (BMI), ASA classification, and Mallampati classification did not differ between the VL-first and DL-first groups.

**Table 1:**
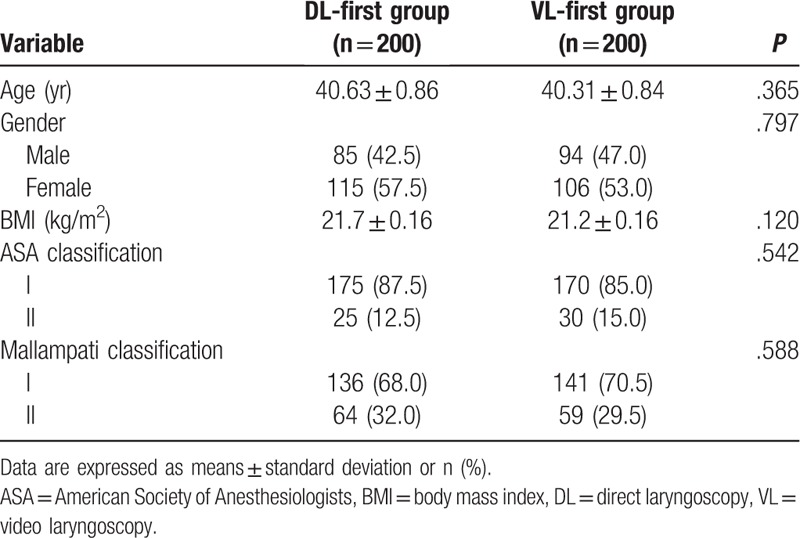
Baseline characteristics of patients in the DL-first and VL-first groups.

### 3.2 Intubation time

Table 2 shows the intubation time of each trainee and mean intubation time of each series. In all, the intubation time of 7 trainees in the second series in the DL-first group and the intubation time of 3 trainees in the second series in the VL-first group were significantly shorter than those in the first series (*P* < .05).

**Table 2:**
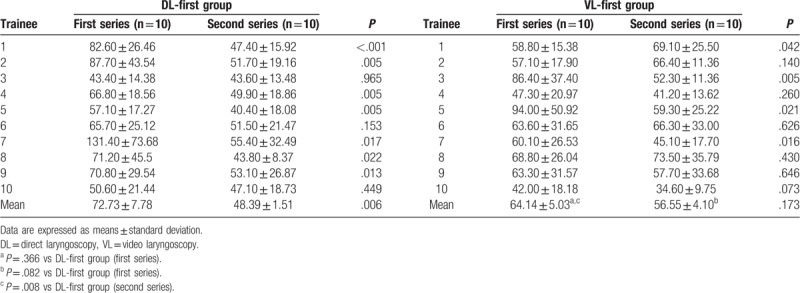
Comparison of intubation time (s) of each trainee in the DL-first and VL-first groups.

Intragroup and intergroup comparisons of the intubation time in the 4 subgroups (DL-first or VL-first groups, each with 2 series) were analyzed, as shown in Table 2. First, the first series of the DL-first and VL-first groups were compared; there were no significant differences in the mean intubation time of the 10 trainees between the 2 groups (*P* *=* .366). Then, the first and second series were compared within the DL-first and VL-first groups. In the DL-first group, the mean intubation time of the second series was significantly shorter (*P* = .006), while in the VL-first group there was no significant difference between the first and second series (*P* = .173). Finally, the second series of the DL-first group was compared to the first series of the VL-first group, and vice versa; the respective *P* were .008 and .082, indicating a significant difference in the mean intubation time between the second series of the DL-first group and first series of the VL-first group, but not between the first series of the DL-first group and second series of the VL-first group.

### 3.3 Learning curve

**Figure 1:**
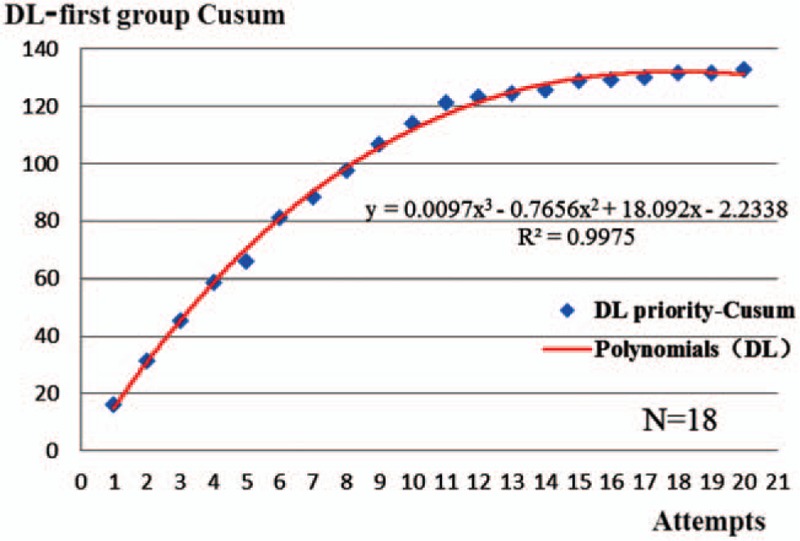
Cumulative summation (CUSUM) chart for ETI. The blue plots are the CUSUM of every attempts of trainees in DL-first group (DL-priority group), and the red line represents the learning curves of trainees (the corresponding formulation: y = 0.0097x^3^–0.7656x^2^+18.092x–2.2338 [R^2^ = 0. 9975]). N means that the mean number of intubation attempts per trainee need exercise for was 18 times in the DL-first group.

**Figure 2:**
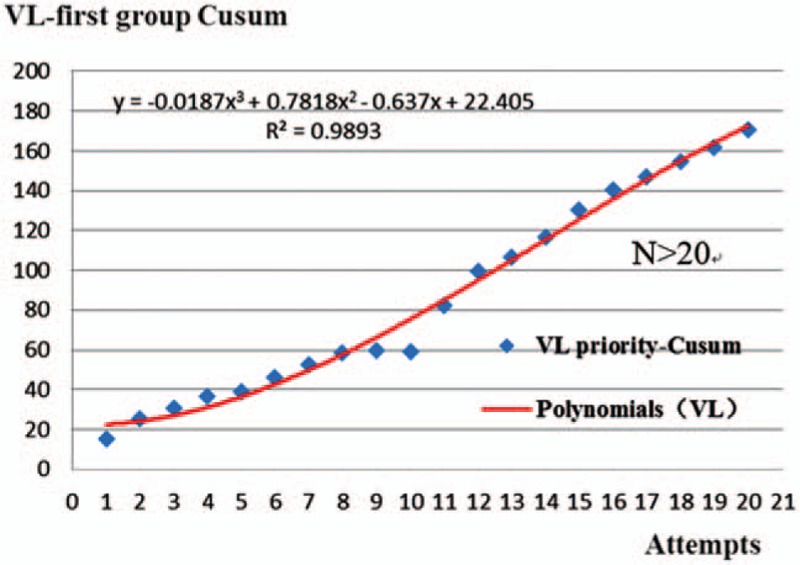
Cumulative summation (CUSUM) chart for ETI. The blue plots are the CUSUM of every attempts of trainees in VL-first group (VL-priority group), and the red line represents the learning curves of trainees (the corresponding formulation: y = –0.0187x^3^ +0.7818x^2^–0.637x+22.405 [R^2^ = 0.9893]). N means that the mean number of intubation attempts per trainee need exercise for was more than 20 times in the VL-first group.

The corresponding graph was plotted according to the CUSUM (Table 3, Figs. 1 and 2). The curve fitting the DL-first group was *y* = 0.0097*x*^3^ – 0.7656*x*^2^ + 18.092*x* – 2.2338 (R^2^ = 0.9975) and that for VL-first group was *y* = –0.0187*x*^3^ + 0.7818*x*^2^ – 0.637*x* + 22.405 (R^2^ = 0.9893). The curve-fitting effects are better for an R^2^ closer to 1. The initial learning process ends at the portion of the curve where the maximum change in slope begins to decrease.^[1]^Table 3 and Figure 1 show the number of attempts needed to cross a 20% acceptable failure rate. The mean number of intubation attempts per trainee was 18 in the DL-first group, and all 10 (100%) trainees crossed the 20% acceptable failure rate line in the CUSUM analysis. More than 20 attempts were required to achieve an 80% ETI success rate for the 10 trainees in the VL-first group (Table 3, Fig. 2). No dental trauma occurred in any patient.

**Table 3:**
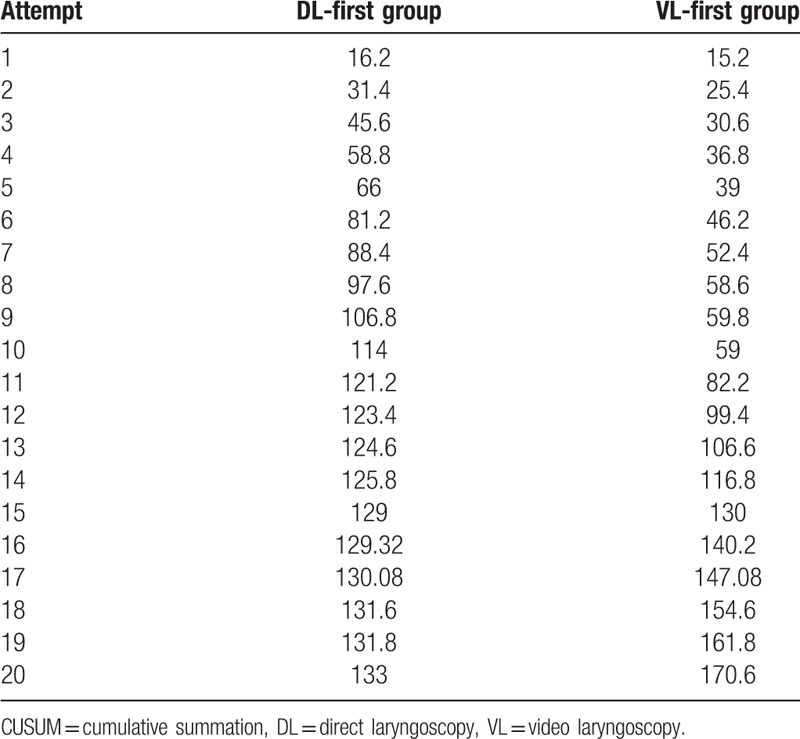
CUSUM of the DL-first and VL-first groups.

### 3.4 Success rates and iatrogenic injury

According to Table 4, the success rates of intubation in the DL-first group (first series), DL-first group (second series), VL-first group (first series), and VL-first group (second series) were 82%, 97%, 81%, and 75%, respectively, while the incidences of adverse events related to intubation in these four subgroups were 33%, 17%, 19%, and 36%, respectively. Therefore, the success rate of intubation with the use of VL in the 2 groups (DL-first and VL-first groups) was significantly higher than for intubation with DL (*P* = .004), while the incidence of adverse events was lower for VL than for DL (*P* < .001). The success rate of intubation in the DL-first group was higher than that in the VL-first group (*P* = .002). The proportions of patients with different CL classifications did not differ significantly between the DL- and VL-first groups (*P* = .083). There was no significant difference in the incidence of adverse events between the DL- and VL-first groups (*P* = .570).

**Table 4:**
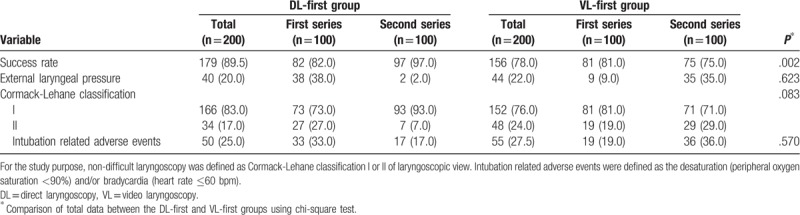
Other recorded data for DL-first and VL-first groups.

## 4 Discussion

Even though many studies have compared the teaching effects of the 2 laryngoscopies,^[5–8]^ no studies have reported whether there is an interaction between DL and VL as teaching tools, that is, whether using DL or VL first could enhance the teaching quality of ETI. Therefore, we examined the influence of the order of teaching ETI through DL and VL on learning by measuring the intubation time and learning curve of trainees, in order to explore ways to improve ETI performance. The results suggest that teaching trainees DL for tracheal intubation first was associated with a better learning performance by shorten the intubation time and reduce the number of attempts.

VL is a newer, more advanced intubation tool than DL and there is no doubt of its superiority when dealing with difficult airways.^[19–21]^ In addition, many studies have proven its advantages for teaching ETI.^[6–8,21]^ Nevertheless, ETI with VL can be more time-consuming because of the need to align the optical axis of the pharynx and mouth and the video laryngoscope might be too big for the patient's mouth. Financial constraints, particularly in developing nations, make substitution of the far more expensive VL devices for the traditional DL blades difficult.^[6]^ Therefore, for the foreseeable future, learning DL will remain an essential skill for future healthcare providers.

The intubation times of 7 trainees in the DL-first group were significantly shorter after performing the first 10 ETIs with DL, while only 3 trainees in the VL-first group had the same results. There was a significant difference in average intubation times for the first and second series in the DL-first group, but not in the VL-first group. This suggests that using DL for ETI first might improve the effects of learning with VL. Our results for the VL-first group support previous studies, such as Nouruzi-Sedeh et al,^[1]^ who found no difference in intubation time between DL and VL. Nevertheless, our results for the DL-first group contradict those of Nouruzi-Sedeh et al We believe that this difference arose because each trainee in the study by Nouruzi-Sedeh et al attempted to intubate only 5 patients with DL and 5 patients with VL, and the number of attempts was insufficient for the trainee to achieve stable intubation time performance.

We found that the average time for ETI in the DL-first group (second series) was significantly shorter than for the VL-first group (first series), while there were no significant differences in the mean intubation time of the DL-first group (first series) and VL-first group (second series). This suggests that using DL as the initial tool for ETI was helpful for reducing the time for ETI. This result contradicts those of Viernes et al,^[6]^ who reported that less time is required for ETI by inexperienced trainees. We could explain the difference in 2 ways. First, VL uses a laryngoscope with a blade with additional upward angulation of the distal half of the blade. This blade is inserted along the midline of the tongue and follows the anatomical upper airway without displacing the tongue and the need to align the optical axes. In contrast to DL, where a Macintosh blade is used, intubation with VL requires that the endotracheal tube be bent with a stylet in almost a U-shape to follow the curve of the blade. This might explain why inexperienced medical students need more time for normal, easy intubations with VL than with DL. Secondly, although Rai et al^[22]^ showed that VL improves the laryngoscopic view by 1 or 2 classifications, the need to align the optical axis of the pharynx and a mouth too small for VL might cause trainees to waste much time coordinating these actions.

Using the CUSUM method, we found that trainees in the DL-first group required 18 procedures to achieve an 80% ETI success rate, while the trainees in the VL-first group required more than 20 attempts. Our results for the DL-first group differ from those of previous studies. Indeed, Komatsu et al^[23]^ found that 29 procedures were required for successful intubation by unexperienced trainees. The criteria for successful tracheal intubation in that study were similar to ours. Our trainees required fewer procedures than in Komatsu et al for 2 reasons. First, we excluded airways that were anticipated to be difficult. Secondly, after the trainees experienced difficulty with DL, they could be more skillful with the use of VL for ETI, because VL, which improves the visualization of the larynx and facilitates intubation, makes up for the deficiencies of DL. In the same line, Low et al^[8]^ showed that trainees using VL had more confidence in their tube placement, and that their success rates and teeth trauma rates were better. Our results for the VL-first group are supported by Toda et al,^[15]^ who found that 30 live experiences of performing an ETI was sufficient for obtaining a 90% ETI success rate. The failure of the VL-first group to improve suggests that using VL as the initial tool for ETI does not lead to a higher ETI success rate. Nair et al^[5]^ showed that in a simulation setting, teaching with VL did not improve learning compared with DL, and that using VL would likely result in the need for more than 2 intubation attempts.

One limitation of our study was that it was conducted at only 1 teaching institution and the number of attempts we allowed to achieve successful intubation might not have been sufficient. Our results might not be generalizable to other teaching institutions, because staff training techniques might differ. In addition, we could not completely eliminate the confounding effects of the type of surgery, anesthetic drugs used, anesthesia depth, individual differences, and other factors. Moreover, the purpose of this article is to explore the teaching order of VL technique and DL technique in ETI for new trainees instead of comparing whether VL technique or DL technique is better for ETI training, thus we compared the “10 VL first + 10 DL later group” with “10 DL first + 10 VL later group”. But we did not show how to learn a designated technique better, for instance, whether it is better to improve DL technique by using “20 DL attempts” or by using “10 VL attempts +10 DL attempts”. Certainly, there is no authoritative criterion for evaluating intubation learning performance. Our study was also based on reducing intubation time and failure rate in the process of learning. Therefore, the results may not be used as authoritative criteria, but only as reference criteria, and further studies will be needed to identify this issue.

## 5 Conclusions

During the teaching of ETI, there was no benefit of using VL first to subsequently learn to perform intubation using DL. By contrast, if trainees first learn to perform ETI using DL, then the subsequent learning of VL will be improved. We consider that trainees be taught to perform DL before learning VL.

## Acknowledgments

Many thanks to all medical students from Nanjing Medical University who participated in this study.

## Author contributions

A Study Design, B Data Collection, C Statistical Analysis, D Data Interpretation, E Manuscript Preparation, F Literature Search, G Funds Collection.

**Conceptualization:** Minglu GU, Lianhua CHEN.

**Data curation:** Minglu GU, Ming LIAN, Chao GONG, Shitong LI.

**Formal analysis:** Minglu GU, Ming LIAN, Chao GONG, Lianhua CHEN, Shitong LI.

**Funding acquisition:** Lianhua CHEN.

**Project administration:** Lianhua CHEN.

**Writing – original draft:** Minglu GU.

**Writing – review & editing:** Ming LIAN, Chao GONG, Lianhua CHEN, Shitong LI.
